# Cervical and oral human papillomavirus infection in women living with human immunodeficiency virus (HIV) and matched HIV-negative controls in Brazil

**DOI:** 10.1186/s13027-020-00301-y

**Published:** 2020-05-11

**Authors:** Tamy Taianne Suehiro, Gabrielle Marconi Zago Ferreira Damke, Edilson Damke, Paloma Luana Rodrigues de Azevedo Ramos, Marcela de Andrade Pereira Silva, Sandra Marisa Pelloso, Warner K. Huh, Ricardo Argemiro Fonseca Franco, Vânia Ramos Sela da Silva, Isabel Cristina Scarinci, Marcia Edilaine Lopes Consolaro

**Affiliations:** 1 Laboratory of Clinical Cytology, Department of Clinical Analysis and Biomedicine, State University of Maringá, Paraná, Brazil; 2 School of Nursing, State University of Maringa, Parana, Brazil; 3 School of Medicine, University of Alabama at Birmingham, Birmingham, AL USA

**Keywords:** HPV, HIV, Oral, Cervical

## Abstract

**Background:**

Despite the demonstrated role of human *Papillomavirus* (HPV) in the etiology of cervical cancer and the strong evidence suggesting the importance of HPV in the development of oropharyngeal cancer, several aspects of the interrelationship between HPV infection in both body sites remain unknown, specifically in female human immunodeficiency virus (HIV)-positive (HIV+) patients. We aimed to assess the prevalence, distribution, and concordance of cervical and oral HPV in HIV+ women and matched HIV-negative (HIV-) controls in Brazil.

**Material and methods:**

Cervical and endocervical samples for cytological screening and HPV detection and oral samples were collected from 115 HIV+ women using highly active antiretroviral therapy (HAART) and 139 HIV-matched controls (HIV-) in Maringá City, Brazil. Risk factors were assessed using a standardized questionnaire, and the data regarding HIV infection were obtained from the patients’ medical records. HPV detection and typing were performed using the Kit Multiplex XGEN Multi HPV Chip HS12.

**Results:**

HIV infection was well controlled in this cohort, but women who exhibited detectable HIV loads were significantly associated with HPV-positive status overall (*P* = 0.03) and in cervical mucosa (*P* = 0.01). HIV+ women had significantly more abnormal cytological findings (*P* = 0.04) than HIV- women. Of the 115 HIV+ women, 48.7% were positive for cervical and/or oral HPV DNA; of the 139 HIV- women, 41% were positive for cervical and/or oral HPV (*P* = 0.25). Both HIV+ and HIV- women had a statistically higher prevalence of cervical HPV infection than oral infection. The concurrent HPV infection in two anatomical sites was similar in HIV+ and HIV- women; however, HPV type concordance was not observed. HPV type distribution was different between the anatomical sites in both groups, and HIV+ women presented less common types, mainly in oral mucosa.

**Conclusion:**

Our data support the importance of testing HPV infection in HIV+ women, even when the HIV infection is well controlled. Prospective studies are required to better understand the natural history of HPV infection in both anatomical sites, specifically in HIV+ women.

## Background

The association between persistent high-risk human *Papillomavirus* (hrHPV), squamous cervical cancer (CC), and some vaginal and anal cancers has been well-established [1–3]. Additionally, recent data demonstrated that HPV is also associated with a subset of head and neck cancers (HNCs), including a worldwide range ~20–80% of oropharyngeal cancers (OPCs) [4, 5]. Individuals living with human immunodeficiency virus (HIV+) are more susceptible to infection, less likely to clear the virus and have a higher risk of HPV-related cancers than HIV-negative (HIV-) individuals [6, 7]. Although the incidence of overall cancer has decreased in HIV+ individuals with the advent of highly active antiretroviral therapy (HAART), HPV-related CC risk is higher among HIV+ individuals [8, 9]. Moreover, in HIV+ women, CC tends to respond poorly to recommended therapies, becomes more aggressive, and may have a worse prognosis [6]. With the increase in survival of HIV+ women due to HAART, progression of oncogenic viral infection into malignancy may result in an increased incidence of HPV-associated oropharyngeal, genital, and anal cancers [8–10].

Currently, the HIV/acquired immunodeficiency syndrome (AIDS) pandemic impacts the poorest and the youngest in low-resource settings [11]. HIV+ young women are key populations at high risk for developing HPV-related cancers, particularly in low- and middle-income countries such as Brazil [12, 13]. With the recent approval of the 9-valent HPV vaccine, the risks of persistent HPV infection and HPV-related cervical precancerous lesions and malignancies are expected to decrease significantly [14]. With the appropriate vaccination rate thresholds, this vaccine provides a logical rationale for increasing screening intervals considering the anticipated decrease in the burden of disease [15]. Therefore, understanding the specific prevalence, distribution and concordance of cervical and oral HPV in at-risk women in specific geographic locations will provide further insight into the effectiveness of this new vaccine against oral HPV infection, which has not been proven yet. Considering the high burden of HPV-related cancers among HIV+ women and the possible effectiveness of a 9-valent HPV vaccine [16], it is critical to understand the prevalence and types of HPV infections in oral and cervical mucosa in HIV+ women (and matched controls). However, while several studies on HIV+ women have reported cervical, oral, or anal HPV type distribution [17–23], only a few studies have addressed concurrent cervical and oral HPV prevalence [24–26]. Despite the demonstrated role of HPV in the etiology of CC and the strong evidence suggesting the importance of HPV in the development of OPC [27–30], several aspects of the interrelationship between oral and cervical infections remain unknown, specifically in HIV+ female patients.

The present study aimed to assess the prevalence, distribution, and concordance of cervical and oral HPV in HIV+ women and matched HIV- controls in the southern region of Brazil, a geographic area with a high incidence of HIV and CC.

## Methods

### Study population

Participants included 115 HIV+ women receiving HAART and 139 HIV- aged 19 to 66 years who attended the Specialized Assistance Service (SAE) for sexually transmitted diseases (STD/AIDS in Maringá, southern Brazil) from September 2017 to May 2018. Women with the following characteristics were included in this study: women with confirmed HIV/AIDS diagnosis using two different methods were included in the HIV+ group and women with two HIV/AIDS negative results using two different methods were included in the HIV- group. Exclusion criteria include: women with previous hysterectomy, pregnant, younger than 19 years, and women with no history of sexual intercourse.

Of the 778 HIV+ women enrolled in the SAE, 324 were eligible for the study. The sample size was calculated with a HPV cervical prevalence of 50% in HIV+ women [19], a 95% confidence interval (CI), and an error estimate of 5%. With an increase of 10% for possible participant losses, the total sample size was fixed at 138 randomly selected women. SAE also provides other services and HIV screening. Therefore, to obtain a comparable sample, 138 matched controls from the list of patients served by the SAE were identified. The controls were matched by age. Written informed consent was obtained from 254 women, and based on their HIV serology status, participants were assigned to the HIV+ (*n* = 115) or HIV- group (*n* = 139). Participants were interviewed using a standardized questionnaire to obtain demographic information (e.g., age, educational attainment, household income, race/ethnicity); tobacco and alcohol use/abuse; obstetric and gynecologic history (e.g., age at menarche, contraceptive use, number of pregnancies); CC screening, and sexual behaviors (e.g., age at first intercourse, number of sexual partners). Data regarding HIV infection and adherence to HAART were obtained from SAE medical records.

### Sample collection

Nursing staff contacted all women, administered the questionnaire, and collected the cervical and oropharyngeal samples. Ecto−/endocervical samples were collected using an Ayre’s spatula and cytobrush for cervical cytology and polymerase chain reaction (PCR) for HPV; the samples for HPV testing were stored in ThinPrep® Pap Test solution. The conventional cytological smears were sent to the Clinical Cytology Laboratory at State University of Maringá (UEM) and were graded according to the Bethesda System [31].

Full-mouth oral/oropharyngeal scraping, including the cheeks, tongue, palate, tonsils, and oropharynx, was performed using a sterile brush with soft bristles; samples were stored in ThinPrep® Pap Test solution. Moreover, a gargle sample was obtained by having the participant gargle with 10 mL of 0.9% sterile saline for a total of 30 s (10s, rinse; 5 s, gargle; 10s rinse; 5 s, gargle), collected in a sterile cup and stored at − 4 °C [20, 32]. All oral/oropharyngeal samples will be referred as “oral samples.”

### HPV DNA testing

Detection and typing of HPV were performed using the Kit Multiplex XGEN MULTI HPV CHIP HS12 (Mobius Life Science), following the manufacturer’s instructions. The multiplex detected the following 36 HPV types: 18 hrHPV (16, 18, 26, 31, 33, 35, 39, 45, 51, 52, 53, 56, 58, 59, 66, 68, 73 e 82) and 18 low-risk HPV (lrHPV) (6, 11, 40, 42, 43, 44, 54, 55, 61, 62, 67, 69, 70, 71, 72, 81, 84 e 89). An additional HPV universal probe was used to detect other, non-specified types of HPV. Samples with invalid outcomes were retested, and the second result was considered definitive. Women were considered positive for oropharyngeal HPV if one of the samples, oral and/or scraping, was positive for HPV. Furthermore, women were considered negative for oropharyngeal HPV if their oral and scraping samples were negative for HPV.

### Statistical analysis

Statistical analyses were performed using the GraphPad Prism 6.0 (San Diego, California, USA) software. All variables were expressed as absolute and relative frequencies. For univariate analysis (unadjusted odds ratios [ORs]), categorical variables were compared with HPV infection using the chi-squared and Fisher’s exact test. Crude ORs and 95% confidence intervals (CIs) were calculated. A *P*-value < 0.05 was considered significant.

## Results

A total of 254 (115 HIV+ and 139 HIV-) women were included in the study, with all 254 cervical and 508 oral samples (oral scraping and gargle) having sufficient DNA for HPV assessment. All 254 (100%) participants had conclusive HPV test results from both anatomical sites.

Demographic and clinical characteristics of the two groups of patients are presented in Table 1. The median age was 42.17 ± 10.18 years old for HIV+ women and 41.4 ± 12.31 years old for HIV- women. Compared to HIV- women, HIV+ women were significantly more likely to have less than 8 years of schooling (*P* = 0.0003), non-white skin color (*P* = 0.009), their first sexual intercourse at < 18 years old (*P* = 0.04), more than two sexual partners (*P* = 0.004 for 2–7 partners and *P* = 0.007 for > 7 partners), and a higher number of parities (*P* = 0.0006 for 1–2 parities and *P* 0.0001 for ≥3 parities), and were less likely to report screening for CC within the past three years (*P* = 0.01). HIV- women were, however, more likely to be a widowed (*P* = 0.004).

**Table 1:** Characteristics of the study population with paired cervical and oral samples, stratified by human immunodeficiency virus (HIV) status

	HIV+ N = 115	HIV- ***N*** = 139	OR (CI)	***P***
	N (%)	N (%)		
**Age cohort (years)**				
**Mean**	42.17	41.4	0.8	0.4
18–30	16 (13.9)	31 (22.3)	1	
31–40	36 (31.3)	37 (26.6)	1.93 (0.88–4.08)	0.09
> 40	63 (54.8)	71 (51.1)	1.72 (0.84–3.36)	0.12
**School education (years)**				
< 8	45 (39.1)	26 (18.7)	2.79 (1.55–5.00)	0.0003
≥ 8	70 (60.9)	113 (81.3)	1	–
**Marital status**				
Married	53 (46.1)	68 (48.9)	1	
Unmarried	45 (39.1)	66 (47.5)	1.14 (0.67–1.95)	0.69
Widowed	17 (14.8)	5 (3.6)	0.22 (0.08–0.61)	0.004
**Skin color**				
White	48 (41.7)	81 (58.2)	1	
Not white	67 (58.2)	58 (41.7)	1.94 (1.17–3.24)	0.009
**Menarche (years)**				
< 13	56 (48.7)	78 (56.1)	1	
≥ 13	59 (51.3)	61 (43.9)	1.34 (0.80–2.18)	0.25
**Age of sexual debut (years)**				
< 18	79 (68.7)	78 (56.1)	1.71 (1.02–2.89)	0.04
≥ 18	36 (31.3)	61 (43.9)	1	
**Sexual partners (number)**				
1	6 (5.2)	24 (17.3)	1	
2–7	77 (66.9)	82 (59)	3.75 (1.43–9.28)	0.004
> 7	32 (27.9)	33 (23.7)	3.87 (1.36–10.68)	0.007
**Parity (number)**				
0	3 (2.6)	26 (18.7)	1	
1–2	59 (51.3)	77 (55.4)	6.64 (2.14–21.50)	0.0006
≥ 3	53 (46.1)	36 (25.9)	12.76 (3.84–41.68)	< 0.0001
**History of cytology in the past three years**				
Yes	42 (36.5)	73 (52.5)	1	
No	73(63.5)	66 (47.5)	1.92 (1.15–3.20)	0.01
**Hormonal contraceptive use**				
Yes	20 (17.4)	37 (26.6)	1	
No	95 (82.6)	102 (73.4)	1.72 (0.93–3.19)	0.08
**Gynecologic infections**				
Yes	30 (26.1)	29 (20.8)	1	
No	85 (73.9)	110 (79.1)	1.33 (0.74–2.40)	0.37
**Cigarette smoking**				
Yes	30 (26.1)	32 (23)	1	
No	56 (48.7)	85 (61.1)	1.2	0.3
Ex-smoker	29 (25.2)	22 (15.8)	0.6	0.1
**HIV diagnosis (years)**				
< 5	73 (63.5)	–	–	–
-10	42 (36.5)	–	–	–
> 10	0 (0)	–	–	–
**HAART compliance**				
Yes	93 (80.9)	–	–	–
No	22 (19.1)	–	–	–
**Current CD4 (**cells/mm^3^)				
< 200	3 (2.6)	–	–	–
200–350	17 (14.8)	–	–	–
> 350	95 (82.6)	–	–	–
**Most recent viral load**				
Undetectable	99 (86.1)	–	–	–
Detectable	16 (13.9)	–	–	–

HAART, highly active antiretroviral therapy; OR = Odds ratio; CI = confidence interval

Most HIV+ women presented excellent control of the HIV infection based on their compliance with HAART (80.9%), preserved CD4+ T lymphocyte count (82.6% with > 350 cells/mm^3^), and suppressed current viral loads (86.1% undetectable). Additionally, most of the HIV+ women had had documented HIV infections for < 5 years (63.5%) (Table 1).

Cytology showed no signs of malignancy in most women from both groups. Overall, 13.0% of HIV+ women and 5.0% of HIV- women presented abnormal cytological findings (*P* = 0.04). Atypical squamous cells of undetermined significance (ASC-US) were observed in 2.6% of HIV+ women and in 1.4% of HIV- women (*P* = 0.66); low-grade squamous intraepithelial lesions (LSIL) were observed in 7.8% of HIV+ women and in 2.8% of HIV- women (*P* = 0.15); high-grade squamous intraepithelial lesions (HSIL) were observed in 2.6% of HIV+ women and 0.8% of HIV- women (*P* = 0.33).

Overall, 56 (48.7%) of the 115 HIV+ women were positive for cervical and/or oral HPV DNA, while 57 (41%) of the 139 HIV- women were positive for cervical and/or oral HPV (*P* = 0.25). Both HIV+ and HIV- women had a statistically higher prevalence of cervical HPV infection than oral infection, including higher rates of hrHPV, lrHPV, universal HPV, and infection by multiple HPV types in cervical samples compared to oral ones, as shown in Table 2.

**Table 2:** Prevalence of human papillomavirus (HPV) status in the cervical and oral mucosa of human immunodeficiency virus (HIV)-positive and HIV-negative women

	Cervical N (%)	Oral N (%)	***P***-value comparing cervical and oral HPV prevalence
**HIV + (n = 115)**			
**Overall**			
Positive	51 (44.4)	17 (14.8)	< 0.0001
Negative	64 (55.6)	98 (85.2)	
**High-Risk HPV**			
Positive	23 (20.0)	7 (6.1)	0.0028
Negative	92 (80.0)	108 (94.0)	
**Low-Risk HPV**			
Positive	37 (32.2)	9 (7.8)	< 0.0001
Negative	78 (67.8)	106 (92.2)	
**Universal HPV**			
Positive	17 (14.8)	7 (6.1)	0.028
Negative	98 (85.2)	108 (93.9)	
**Multiple types Infections**			
Positive	28 (24.3)	5 (4.4)	< 0.0001
Negative	87 (75.7)	110 (95.6)	
**HIV - (n = 139)**			
**Overall**			
Positive	52 (37.4)	13 (9.4)	< 0.0001
Negative	87 (62.6)	126 (90.6)	
**High-Risk HPV**			
Positive	33 (23.7)	5 (3.6)	< 0.0001
Negative	106 (76.3)	134 (96.4)	
**Low-Risk HPV**			
Positive	29 (20.9)	5 (3.6)	< 0.0001
Negative	110 (79.1)	134 (96.4)	
**Universal HPV**			
Positive	22 (15.8)	4 (2.9)	0.0003
Negative	117 (84.2)	135 (97.1)	
**Multiple types infections**			
Positive	29 (20.9)	4 (2.9)	
Negative	110 (79.1)	135 (97.1)	< 0.0001

HPV DNA was detected in oral samples from 17 (14.8%) HIV+ women and 13 (9.4%) HIV- women (*P* = 0.24) (Table 3). Multiple HPV infections were detected in five samples (4.4%) from HIV+ women and in four samples (2.9%) from HIV- women (Table 2). Statistical analysis did not reveal an association between HIV+ status and the presence of HPV DNA in the oral mucosa (*P* = 0.83).

**Table 3:** Detection of human papillomavirus (HPV) infection in the oral mucosa and uterine cervix of human immunodeficiency virus (HIV) + and HIV- women

	HIV+ (N = 115) N (%)	HIV- (N = 139) N (%)	***P***
Oral mucosa	17 (14.8)	13 (9.4)	0.24
Uterine cervix	51 (44.3)	52 (37.4)	0.48
Positive samples from oral mucosa of patients with infected uterine cervix	8 (7.0)	7 (5.0)	0.6
Positive samples from oral mucosa of patients with uninfected uterine cervix	3 (2.6)	5 (3.6)	0.73

Fifty-one (44.3%) HIV+ women and 52 (37.4%) HIV- women had HPV-positive cervical samples (*P* = 0.48) (Table 3). Multiple HPV types were detected in 28 (24.3%) of HIV+ and in 29 (20.9%) of HIV- patients (Table 2). There was no significant difference between the HIV+ and HIV- women with regard to HPV types.

To better understand the association between cervical and oral HPV infection, the prevalence of concurrent HPV infection in this population was investigated. Eight (7.0%) HIV+ women and 7 (5.0%) HIV- women had concurrent HPV infection in their cervical and oral samples (*P* = 0.6) (Table 3). Concordance between HPV types in the cervical and oral samples was not observed in either group.

**Fig. 1:**
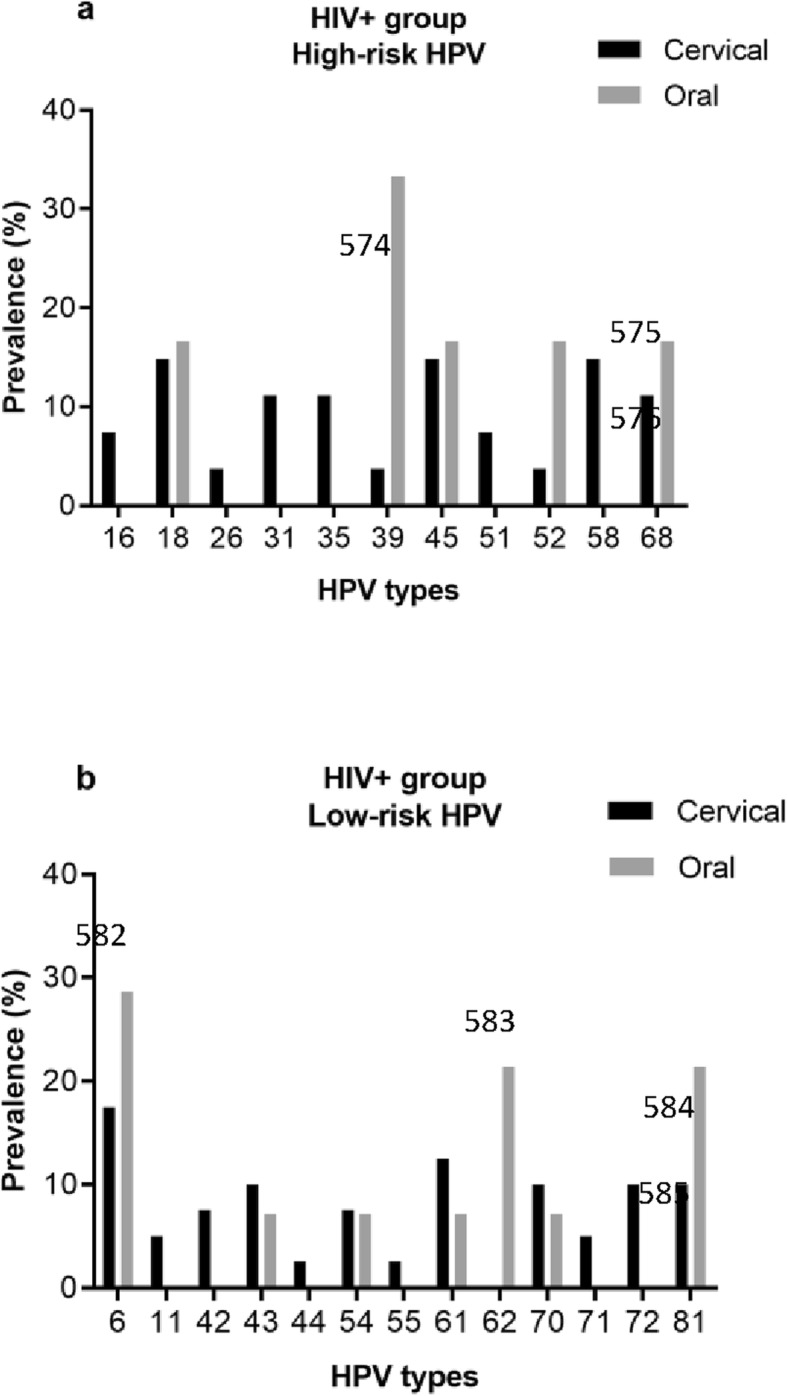
Human *Papillomavirus* (HPV) type distribution of cervical and oral infections detected in the human immunodeficiency virus-positive group. **a** High-risk HPV type distribution. **b** Low-risk HPV type distribution

In the HIV+ group, the most frequent cervical hrHPV types observed were HPV18, HPV45, and HPV58 (14.8% each). In the oral site, the most prevalent hrHPV was HPV39 (33.3%) followed by HPV18, HPV45, HPV52, and HPV68 (16.6% each). The most prevalent cervical lrHPV found in this group was HPV6 (17.5%), followed by HPV61 (12.5%), and in oral mucosa, the most prevalent lrHPV was also HPV6 (28.6%), followed by HPV62 and HPV81 (21.4%) (Fig. 1).

**Fig. 2:**
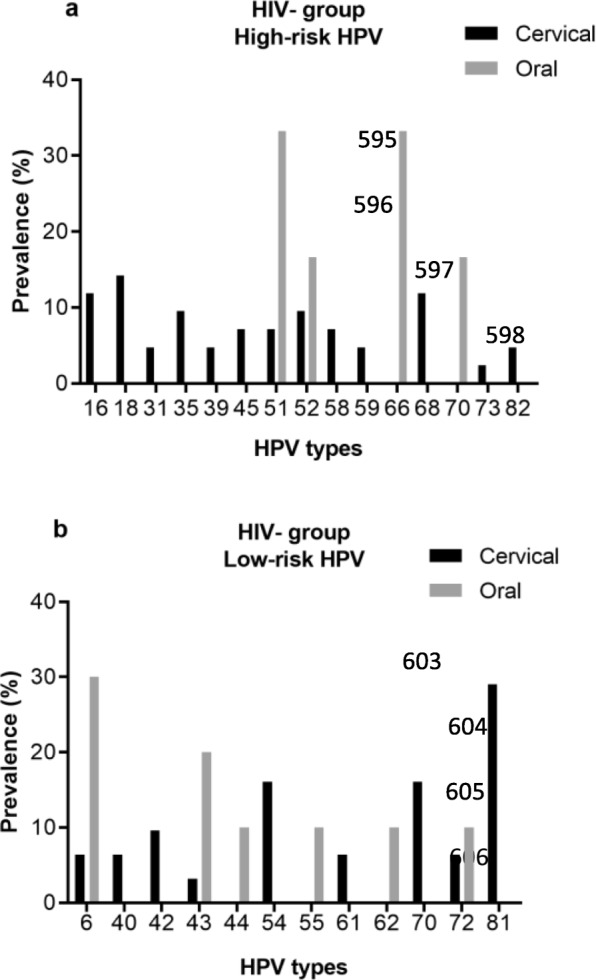
Human *Papillomavirus* (HPV) type distribution of cervical and oral infections detected in the human immunodeficiency virus-negative group. **a** High-risk HPV type distribution. **b** Low-risk HPV type distribution

In the HIV- group, the most prevalent hrHPV in the cervical mucosa was HPV18 (14.2%), followed by HPV16 and HPV68 (11.9% each). In the oral site, HPV51 and HPV66 were the most prevalent hrHPV (33.3% each). The most prevalent lrHPVs in the cervical site were HPV81 (29.0%) and HPV54 and HPV70 (16.1% each), and the most prevalent lrHPV in the oral site was HPV6 (30.0%), followed by HPV43 (20%) (Fig. 2).

HIV+ women with recent detectable HIV loads were significantly associated with HPV-positive status overall (OR = 3.75; CI = 1.22–11.10; *P* = 0.03) and in cervical mucosa (OR = 4.61; CI = 1.50–13.66; *P* = 0.01) (Table 4).

**Table 4:** Prevalence of cervical and oral HPV infection according to the HIV-related parameters

Variables	HPV+ (***N*** = 56)	OR (CI)	***P***	Cervical HPV+ (***N*** = 51)	OR (CI)	***P***	Oral HPV+ (*N* = 11)	OR (CI)	***P***
	N (%)			N (%)			N (%)		
**Documented HIV infection (years)**									
< 5	39/73 (53.4)	1.68 (0.79–3.60)	0.24	36/73 (49.3)	1.75 (0.81–3.71)	0.18	7/73 (9.6)	1.00 (0.28–3.23)	> 0.99
5–10	17/42 (40.5)	1	–	15/42 (35.7)	1		4/42 (9.5)	1	
> 10	0/0 (0)	–	–	–	–		–	–	–
**Compliance to HAART**									
Yes	42/93 (45.2)	0.46 (0.18–1.17)	0.15	39/93 (41.9)	0.60 (0.22–1.50)	0.34	9/93 (9.7)	1.07 (0.23–5.25)	> 0.99
No	14/22 (66.6)	1	–	12/22 (54.5)	1		2/22 (9.1)	1	
**Most recent CD4 (cells/mm**^**3**^**)**									
< 200	3/3 (100)	–	–	3/3 (100)	–	–	0/3 (0)	–	–
200–350	9/17 (52.9)	1.30 (0.47–3.78)	0.79	8/17 (47.1)	1.22 (0.41–3.34)	0.79	2/17 (11.8)	1.27 (0.25–6.01)	0.67
> 350	44/95 (46.3)	1	–	40/95 (42.1)	1		9/95 (9.5)	1	
**Most recent viral load**									
Undetectable	44/99 (44.4)	1	–	39/99 (39.4)	1		11/99 (11.1)	1	–
Detectable	12/16 (75.0)	3.75 (1.22–11.10)	0.03	12/16 (75.0)	4.61 (1.50–13.66)	0.01	0/16 (0)	–	

HPV = human papillomavirus, HIV = human immunodeficiency virus, HAART, highly active antiretroviral therapy; OR = Odds ratio; CI = confidence interval

When the characteristics of the HIV+ women were analyzed in relation to HPV status, only current smoking was associated with overall (OR = 3.80; CI = 1.22–10.42; *P* = 0.01) and cervical HPV-positive status (OR = 3.94; CI = 1.31–11.76; *P* = 0.01 for both), as shown in Table 5.

**Table 5:** Possible predictors of cervical and oral human papillomavirus (HPV) infection, stratified by HPV status in human immunodeficiency virus (HIV)-positive women

Predictors of HPV infection	HPV+ (N = 56)	OR (CI)	***P***	Cervical HPV+ (N = 51)	OR (CI)	***P***	Oral HPV+ (N = 11)	OR (CI)	***P***
	N (%)			N (%)			N (%)		
**Age (years)**									
18–29	7/13 (58.4)	1	–	7/13 (58.3)	1		0/13 (0)	–	–
30–50	35/76 (46.1)	0.60 (0.19–2.01)	0.53	30/76 (39.5)	0.46 (0.15–1.54)	0.34	8/76 (10.5)	1	
> 50	14/27 (51.8)	0.76 (0.20–3.02)	0.74	14/27 (51.8)	0.76 (0.19–3.03)	0.74	3/27 (11.1)	1.06 (0.26–4.33)	> 0.99
**Race**									
White	24/48 (50.0)	0.54 (0.16–1.69)	0.39	22/48 (45.8)	0.46 (0.14–1.45)	0.26	6/48 (12.5)	2.28 (0.25–20.51)	0.66
Brown	21/49 (42.9)	0.40 (0.12–1.26)	0.16	18/49 (36.7)	0.31 (0.09–0.99)	0.05	4/49 (8.2)	1.42 (0.14–13.70)	> 0.99
Black	11/17 (64.7)	1	–	11/17 (64.7)	1		1/17 (5.9)	1	
Asian	0 (0)	–	–	0 (0)	–	–	0 (0)	–	–
**Sexual debut (years)**									
< 16	22/45 (48.9)	1	–	21/45 (46.7)	1		4/ (8.9)	1	
≥ 16	34/70 (48.6)	0.98 (0.47–2.06)	1	30/70 (42.9)	1.16 (0.55–2.44)	0.70	7/70 (10.0)	1.14 (0.31–4.13)	> 0.99
**Smoking status**									
Never smoker	26/56 (46.4)	1.64 (0.62–4.08)	0.35	25/56 (44.6)	2.11 (0.80–5.58)	0.16	3/56 (5.4)	0.49 (0.09–2.60)	0.40
Current smoker	20/30 (66.7)	3.80 (1.22–10.42)	0.01	18/30 (60.0)	3.94 (1.31–11.76)	0.01	5/30 (16.7)	1.73 (0.38–7.02)	0.70
Ex-smoker	10/29 (34.5)	1	–	8/29 (27.6)	1		3/29 (10.3)	1	
**Lifetime number of sexual partners**									
< 5	25/51 (49.0)	1	–	25/51 (49.0)	1		5/51 (9.8)	1	
≥ 5	31/64 (48.4)	0.97 (0.48–1.98)	1	26/64 (40.6)	0.71 (0.34–1.50)	0.45	6/64 (9.4)	1.19 (0.31–4.46)	> 0.99
**Use of hormonal Contraceptives**									
Yes	11/20 (55.0)	1	–	9/20 (45.0)	1		2/20 (10.0)	1	
No	45/95 (47.4)	0.73 (0.29–1.98)	0.9	42/95 (44.2)	0.96 (0.36–2.55)	> 0.99	9/95 (9.5)	0.94 (0.18–4.73)	> 0.99

OR = Odds ratio; CI = confidence interval

## Discussion

In the present study, we aimed to determine the HPV prevalence, distribution, and type concordance between cervical and oral samples of HIV+ women and HIV- matched controls in the southern region of Brazil, a geographic area with high incidences of HIV and CC. Our data demonstrated that HIV+ and HIV- women had a similar and high HPV prevalence in cervical and in oral sites. However, HIV+ women had higher prevalence of abnormal cytological findings than HIV- women. HPV type distribution was different between the anatomical sites within both groups, and HIV+ women commonly presented with narrower HPV type distributions, mainly in the oral mucosa. Finally, the frequency of concurrent HPV infection in both sites was low, and HPV type concordance was not observed.

Our results showed that the prevalence of cervical HPV infection was 44.3% in HIV+ women and 37.4% in HIV- women. Studies across different populations present varying rates of HPV infection: 45–97.1% in HIV+ women and 44.9–86.5% in HIV- women in the cervical site [20, 24, 25, 33]. The high prevalence of HPV in HIV-negative women in this study can be explained, at least in part, by the fact they were recruited from a Specialized Assistance Service (SAE) for sexually transmitted diseases, and were therefore at higher risk for sexually transmitted infections, including HPV. Additionally, the rates of oral HPV infection in HIV+ women and HIV- control were 14.8 and 9.4%, respectively. Other studies have shown that the prevalence of HPV infection in the oral site can vary significantly (12–68.5% in HIV+ women and 2–31.4% in HIV- women) [20, 24, 33].

Although HIV infections were well controlled and cytology results showed no malignancies, abnormal cytological findings were significantly higher in HIV+ women than in HIV- women. These data are consistent with the results of the previous studies showing that HIV+ women are less likely to eliminate the virus, have subsequent persistent hrHPV infections and have higher risk of developing precancerous lesions and malignancies, even with the appropriate use of antiretroviral therapy, than HIV- women [8, 9, 19, 34, 35].

Our results demonstrated no significant difference in HPV detection in the uterine cervix or oral mucosa between HIV+ women and HIV- women; however, HPV infection was significantly higher in the cervical mucosa than in the oral site in both groups. These findings are consistent with previous studies suggesting that the natural history of HPV infection varies by anatomical site, and a higher prevalence of HPV infection is observed in the cervical mucosa than in the oral mucosa in HIV+ women [36–38]. This can be explained, at least in part, by the evidence that the oral cavity is a hostile environment for the establishment of infectious agents due to the presence of both mechanical and molecular mechanisms related to digestion [39]. More specifically, Fakhry et al. [39] conducted a study that directly compared the local immunologic profiles of the oral cavities and cervices of healthy women using paired secretion specimens. This study showed that the oral cavity contained significantly higher concentrations of immunoregulatory factors that were related to the adaptive and cell-mediated immune response than the cervix, which may in part explain the significantly lower burden of sexually transmitted infections such as *Chlamydia trachomatis*, HPV, and HIV-1 in the oral cavity than in the cervix. According to the authors, these findings provide additional information to better understand the differences in the etiology and natural history of pathogenic agents that are capable of colonizing both the oral cavity and female reproductive tract.

The most prevalent HPV types in the cervical samples from both HIV+ and HIV- women were hrHPV18 and hrHPV58; however, hrHPV45 and hrHPV16 were frequently detected in HIV+ women and HIV- women, respectively. Prevalence studies around the world have shown that hrHPV types 16, 18, 31, 33, 35, 52, and 58 are the most commonly detected hrHPVs in CC, with hrHPV16 being the most common in all populations, with the exception of HIV+ people [1]. Data have consistently shown that HIV+ women are frequently more infected with other hrHPV types than hrHPV16 and hrHPV18, such as hrHPV52 and hrHPV58 [40]. The high prevalence of non-vaccine hrHPV types of 2-valent and 4-valent vaccines in the cervical and oral mucosa found in our study suggests that the 9-valent HPV vaccine is significantly required, which is considered important to reduce the risk of developing HPV-related cancers, specifically in the HIV+ population.

Unlike the distribution of HPV types in cervical samples, HPV type concordance between the HIV+ and HIV- groups was not observed in oral samples. hrHPV39 was the most common hrHPV detected in oral samples of HIV+ women, followed by hrHPV18, hrHPV45, hrHPV52, and hrHPV68, with these types being also frequently found in cervical samples in the same group. However, in HIV- women, the oral hrHPV types observed were totally different, with hrHPV51 and hrHPV66 being the most frequently detected hrHPV. Current evidence has shown that hrHPV16 and hrHPV18 contribute to the majority (approximately 85%) of HNC cases worldwide, while the remaining cancers are caused by hrHPV33, hrHPV35, hrHPV52, hrHPV45, hrHPV39, and hrHPV58 [41, 42].

Examination of concurrent HPV infections in different anatomical sites has been limited. Hence, we concurrently investigated the prevalence of HPV infection and HPV type distribution in cervical and oral sites to better understand their clinical significance. Simultaneous HPV cervical and oral infection was low in both HIV+ and HIV- women. Moreover, HPV type concordance was not observed, a finding consistent with previous studies [20, 24]. Taken together, these observations suggest that various HPV types are more likely to occur in a different anatomical sites, and/or that the two anatomical sites can clear certain HPV types or distinct exposures, while allowing other HPV types to cause persistent oral and cervical HPV infections.

Prior investigations have demonstrated that engagement in some high-risk behaviors may facilitate HPV infection and act as a cofactor for viral persistence, contributing to cancer progression in the oral and cervical mucosa. In the present study, we found that current smoking was associated with overall and cervical HPV infection in HIV+ women. Several studies have already demonstrated that smoking status acts as a predictor and cofactor in cervical HPV infection, which is possibly due to the alteration of the mucosa cells, making them more susceptible to infection, changing the immune mediators, causing DNA damage, and promoting the integration of HPV DNA into the host genome [42–45].

## Conclusion and future perspectives

Our study demonstrated that HIV+ and HIV- women in southern Brazil had high HPV prevalence in cervical and oral sites. In this population, HIV+ women had more abnormal cytological findings than HIV- women. This study provides important epidemiological data about the potential risk of developing CC. Furthermore, the high prevalence of non-vaccine hrHPV types of the 2-valent and 4-valent vaccines in the cervical and oral mucosa of HIV+ and mainly in HIV- women found in our study highlights the importance of the 9-valent vaccine. Concurrent HPV infection in both sites was uncommon, and HPV type concordance was not observed, likely reflecting the differences in the risk factors and the natural history of HPV infection at the two anatomical sites. These findings confirm the prevalence of HPV infection in HIV+ women. Prospective studies are required to better understand the natural history of HPV infection in both anatomical sites, specifically in HIV+ women, and the impact of vaccination programs in at-risk groups.

## Data Availability

All data are included in the manuscript.

## Abbreviations

ASC-US: squamous cells of undetermined significance
CC: squamous cervical cancer
CI: confidence interval
HAART: highly active antiretroviral therapy
HIV: human immunodeficiency virus
HNCs: head and neck cancers
HPV: human *Papillomavirus*
hrHPV: high-risk HPV
HSILs: high-grade squamous intraepithelial lesions
lrHPV: low-risk HPV
LSILs: low-grade squamous intraepithelial lesions
OPCs: oropharyngeal cancers
OR: odds ratio
Pap: Papanicolaou
SAE: Specialized Assistance Service
